# Availability of Oral Emergency Contraception in German Community Pharmacies—A Nationwide Cross‐Sectional Mystery Caller Study

**DOI:** 10.1002/hsr2.72050

**Published:** 2026-03-15

**Authors:** Christian Kunow, Carolin Priepke, Bernhard Langer

**Affiliations:** ^1^ Department of Health, Nursing, Management Neubrandenburg University of Applied Sciences Neubrandenburg Germany

**Keywords:** availability, community pharmacy services, levonorgestrel, mystery calls, patient simulation, ulipristal acetate

## Abstract

**Background and Aims:**

In Germany, the “morning after pill” (oral emergency contraception [EC]), which contains the active ingredients ulipristal acetate (UPA) and levonorgestrel (LNG), can only be dispensed by community pharmacies (CPs). Consequently, German CPs bear significant responsibility with regard to the availability of oral EC, which is an important criterion for immediate and unhindered access. This study aimed to investigate the availability of oral EC in CPs nationwide for the first time in the world. In addition, the influence of possible factors on availability was examined.

**Methods:**

This cross‐sectional study was based on the simulated patient methodology, which is considered the “gold standard”. Each of the 392 CPs was called once by one of six trained mystery callers (MCs), representing a nationwide sample. At the beginning of the conversation, the MCs asked about the “morning after pill” without naming a specific oral EC. In the conversation about UPA (scenario‐related appropriate outcome due to unprotected sexual intercourse 4 days ago) or LNG preparations (scenario‐related inappropriate outcome), the MCs asked about the respective immediate availability.

**Results:**

Three hundred and ninety‐two planned mystery calls were successfully carried out. Information on the availability of UPA preparations was obtained in 318 mystery calls, and of LNG preparations in 38 mystery calls. UPA preparations were immediately available in 97.5% (310/318) and LNG preparations in 100% (38/38) of the CPs. None of the investigated factors had a significant influence on availability.

**Conclusion:**

While oral EC is generally immediately available in Germany, the optimal rate of 100% immediate availability was not reached for UPA preparations, which may hinder access.

## 1 Introduction

To prevent an unwanted pregnancy following unprotected sexual intercourse (UPSI), such as condom failure, the World Health Organization (WHO) recommends the use of emergency contraception (EC) [1]. The best‐known EC in Germany is the “morning‐after pill” (oral EC) [2]. Oral EC is only approved as a single‐dose preparation containing either ulipristal acetate (UPA) or levonorgestrel (LNG) [3]. For both active ingredients, there are numerous generics available in Germany in addition to the originals [4], with no statutory age restriction and, according to product information, for all women of reproductive age [5]. The effectiveness of LNG is significantly lower compared to UPA among individuals with obesity [6]. At 120 h after UPSI, UPA has a longer effectiveness compared to LNG (72 h) and is more effective in the first 24 or 72 h after UPSI [7]. Due to this short window of effectiveness, low access barriers, such as immediate availability, are especially important for UPA products. This is crucial because oral EC is most effective when taken as soon as possible after UPSI [1].

The WHO classifies oral EC as an essential medicine [8], which should be immediately available and stocked in sufficient quantities [9]. In Germany, oral EC—similar to many countries worldwide [10]—is also available as an over‐the‐counter (OTC) medicine without a prescription and may only be dispensed by community pharmacies (CPs). With approximately 17,000 CPs and about 900,000 packages of oral EC dispensed annually in Germany [11], each CP, regardless of location (rural or urban), dispenses oral EC on average once per week. German CPs, therefore, play a key role in ensuring immediate access to oral EC. However, customers can only obtain information on availability from the CPs themselves, not from any other sources. This raises the question of whether CPs in Germany have oral EC immediately available. While there are already nationwide studies on availability in CPs (e.g., [12]), none have specifically focused on oral EC. Therefore, the aim of the present study was to investigate the availability of oral EC in German CPs nationwide.

## 2 Methods

### 2.1 Design

This cross‐sectional study was based on the simulated patient methodology (SPM) as a form of covert participatory observation, which is internationally frequently used [13] and considered the “gold standard” [14, 15]. Here, a seemingly real customer contacts a CP and simulates participation in a seemingly real service process based on a scenario [13, 14]. Since SPM in the form of visits is associated with considerable effort for large sample sizes [14], the study was based on SPM in the form of calls (“mystery calls”), which has already been used frequently in the CP setting (e.g., [16, 17, 18, 19, 20, 21, 22, 23, 24, 25, 26, 27, 28, 29, 30, 31, 32, 33]). The SPM study in the form of calls was reported in accordance with the “STROBE Statement” [34]. Extensive information on the method can be found in the published study protocol [35].

### 2.2 Mystery Callers (MCs)

The mystery calls were performed by six trained individuals, referred to as MCs, from August to October 2021. The MCs, all of German ethnicity, included one former student (38 years old) as well as one male and four female students (aged between 20 and 30 years) from the Department of Health, Nursing, Management at the Neubrandenburg University of Applied Sciences, within the framework of a three‐semester research project of various Master's degree programs. The five students were within the typical age range of average users of oral EC in Germany [36]. None of the MCs had prior experience as MCs. Participation in the research project was unpaid.

### 2.3 Setting and Participation

This MC study used a representative random sample of German CPs stratified according to the 16 federal states (minimum sample size *n* = 392). The allocation of the 392 CPs to the MCs was carried out randomly, resulting in each of the six MCs being assigned 65 or 66 CPs. The mystery calls were conducted on different weekdays and at varying times of day. In addition, 3 months prior to the start of the main study, each MC conducted five validation calls to CPs outside the random sample as part of a pilot study. The total of 30 validation calls showed that no adjustments to the planned scenario or to the assessment items were necessary.

### 2.4 Scenario and Assessment

This MC study was based on a conversation situation as close to real life as possible and used a product‐based scenario. At the beginning of the conversation, the MCs asked about the “morning after pill” without naming a specific oral EC. From that point on, the pharmacy staff could end the conversation at any time and, for example, refer the supposedly affected person to a visit to the CP in person or to a doctor's visit. In the conversation about UPA (scenario‐related appropriate outcome due to a condom failure 4 days ago) or LNG preparations (scenario‐related inappropriate outcome), the MCs inquired about the respective immediate availability. If the pharmacy staff denied availability, the MCs asked when the respective preparation would be available. If the pharmacy staff voluntarily indicated which other CP had the respective oral EC available, this information was also collected. The data collection was completed by recording possible influencing factors (Table 1).

**Table 1:** Possible influencing factors, time, and type of data collection.

Possible influencing factors [literature sources]	Time of data collection	Type of data collection
Call attempts [37]	During the mystery call(s)	Exact measurement by the number of call attempts
Time of the mystery call [25]	During the mystery call	Exact measurement using the watch of the MC
Call duration [22]	During the mystery call	Exact measurement based on the display of the phone of the MC
Gender of the MCs [28]	Before the mystery call	Exact measurement based on the gender of the MC
MC themselves [38]	Before the mystery call	Exact measurement by linking the CPs to the appropriate MCs
Gender of the pharmacy staff [17]	During the mystery call	Estimate by acoustic impression of the MC
CP quality certificate [39]	After the mystery call	Exact measurement using a telephone query by the MCs after completing all the mystery calls
Degree of urbanization [40, 41] of the CPs [29]	After the mystery call	Exact measurement using internet research
Purchasing power index [42] by city/district of the CPs [27]	After the mystery call	Exact measurement using internet research

### 2.5 Data Collection

For data collection, the MCs attempted to reach the respective CP a maximum of three times within a single day using their private mobile phones with the caller ID suppressed. If a CP could not be reached, the MCs marked the CP as unreachable and selected another CP within the corresponding stratum. For quality assurance, a second observer was involved, who listened to the mystery call via the speakerphone or conference call function of the MCs' mobile phones. The MCs collected the data immediately after the mystery call with the help of the second observer. Any disagreements were resolved through discussion between the respective MC and the second observer. If no agreement could be reached, the assessment of the second observer was decisive.

### 2.6 Data Management and Analysis

Data were entered using the four‐eyes principle and analyzed with SPSS Version 31 for Windows (IBM, Armonk, NY, USA). As part of the descriptive statistics, frequencies and percentages were calculated. Associations between the immediate availability of UPA preparations and possible influencing factors were examined using Fisher's exact test [43] or the Fisher–Freeman–Halton exact test [44], as appropriate. Due to the 100% immediate availability of LNG preparations, an analysis of associations with possible influencing factors was not applicable. Cramer's V was reported as a measure of effect size. In all analyses, a two‐tailed *p* value of less than 0.05 was considered statistically significant.

### 2.7 Ethical Considerations

The study protocol was approved by the institutional ethics committee of Neubrandenburg University of Applied Sciences (registration number: HSNB/171/21). The mystery calls were conducted in a covert manner, thereby precluding CPs from opting out in order to circumvent both a Hawthorne effect and a selection bias. Participant‐informed consent was waived by the institutional ethics committee due to the specific study design (covert participant observation) required to achieve the research objectives. To address the CPs' need for information, a letter was sent approximately 6 months prior to the data collection period, providing them with pertinent details. However, in order to avoid compromising the concealed study design, the letter specified a correspondingly long period (“calls are planned from August to October 2021”) rather than a specific date. The data collected was recorded, stored, and processed in an anonymous manner, without the use of audio recordings. Furthermore, the authors and MCs have provided written assurances that all collected experiences and data will be treated as strictly confidential. In addition, recruited students provided their written informed consent to act as MCs.

## 3 Results

Of the 392 CPs, 10 could not be reached by phone even after three call attempts and were replaced with other CPs from the respective federal states, resulting in a total of 402 CPs being called. This ensured that all 392 planned mystery calls were successfully completed (call completion rate: 100%). Nearly four‐fifths of the CPs (86.0%, 337/392) were reached on the first attempt. The mystery calls were distributed fairly evenly throughout the day, with minor peaks in the late morning (39.8%, 156/392) and early evening (37.5%, 147/392). In most cases, the call duration was under 1 min and 30 s. About two‐thirds of the mystery calls were conducted by female MCs (66.6%, 261/392). The pharmacy staff were also female in most cases (88.3%, 346/392). Where determinable, the proportion of CPs with (25.5%, 100/392) and without (26.8%, 105/392) a quality certificate was nearly balanced. With regard to the degree of urbanization, the CPs called were predominantly located in cities (40.0%, 157/392) as well as towns and semi‐dense areas (39.3%, 154/392). The CPs contacted were primarily (58.4%, 229/392) located in cities or districts with below‐average purchasing power (Table 2).

**Table 2:** Characteristics of the mystery calls, the CPs, pharmacy staff, and MCs.

		Frequency (*n*) Total (392)	Percentage (%) Total (100)
Call attempts	1 call attempt	337	86.0
	2 call attempts	43	11.0
	3 call attempts	12	3.0
Time of the mystery calls	08:00 a.m.−12:00 p.m.	156	39.8
	12:01 p.m.−04:00 p.m.	89	22.7
	04:01 p.m.−08:00 p.m.	147	37.5
Call duration	≤ 1:30 min	206	52.6
	1:31−3:00 min	149	38.0
	> 3:00 min	37	9.4
Gender of the MCs	Female	261	66.6
	Male	131	33.4
MC themselves	1	66	16.8
	2	65	16.6
	3	65	16.6
	4	65	16.6
	5	66	16.8
	6	65	16.6
Gender of the pharmacy staff	Female	346	88.3
	Male	46	11.7
CP quality certificate	Yes	100	25.5
	No	105	26.8
	Not able to be determined	187	47.7
Degree of urbanization of the CPs	Cities	157	40.0
	Towns and semi‐dense areas	154	39.3
	Rural areas	81	20.7
Purchasing power index by city/district of the CPs	≤ 100%	229	58.4
	> 100%	163	41.6

Information on the availability of UPA preparations was obtained in 318 mystery calls, and of LNG preparations in 38 mystery calls. UPA preparations were immediately available in 97.5% (310/318) and LNG preparations in 100% (38/38) of the CPs. 25.0% (2/8) of the CPs who did not have UPA immediately available voluntarily provided the MCs with information about whether another CP had UPA available. Upon request, 50.0% (4/8) of the CPs who did not have UPA immediately available stated that UPA would be available later the same day, while the remaining 50.0% (4/8) indicated availability the following day. No significant associations were found between the immediate availability of UPA preparations and the possible influencing factors (Table 3).

**Table 3:** Possible influencing factors on the immediate availability of UPA preparations.

Possible influencing factors	Total	Immediate UPA availability	Immediate UPA non‐availability	*p* value (effect size)
	*n* (%) 318 (100)	*n* (%) 310 (97.5)	*n* (%) 8 (2.5)	
Call attempts				
1 call attempt	271 (100)	266 (98.2)	5 (1.8)	0.11 (0.13)^a^
2 call attempts	36 (100)	33 (91.7)	3 (8.3)	
3 call attempts	11 (100)	11 (100)	0 (0.0)	
Time of the mystery calls				
08:00 a.m.−12:00 p.m.	128 (100)	126 (98.4)	2 (1.6)	0.53 (0.06)^a^
12:01 p.m.−04:00 p.m.	74 (100)	71 (95.9)	3 (4.1)	
04:01 p.m.−08:00 p.m.	116 (100)	113 (97.4)	3 (2.6)	
Call duration				
≤ 1:30 min	149 (100)	145 (97.3)	4 (2.7)	0.27 (0.08)^a^
1:31−3:00 min	135 (100)	133 (98.5)	2 (1.5)	
> 3:00 min	34 (100)	32 (94.1)	2 (5.9)	
Gender of the MCs				
Female	204 (100)	200 (96.5)	4 (3.5)	0.46 (0.05)^b^
Male	114 (100)	110 (98.0)	4 (2.0)	
MC themselves				
1	52 (100)	52 (100)	0 (0)	0.42 (0.13)^a^
2	53 (100)	50 (94.3)	3 (5.7)	
3	47 (100)	47 (100)	0 (0)	
4	58 (100)	56 (96.6)	2 (3.4)	
5	56 (100)	54 (96.4)	2 (3.6)	
6	52 (100)	51 (97.5)	1 (2.5)	
Gender of the pharmacy staff				
Female	284 (100)	276 (97.2)	8 (2.8)	> 0.99 (0.06)^b^
Male	34 (100)	34 (100)	0 (0)	
CP quality certificate				
Yes	89 (100)	88 (98.9)	1 (1.1)	0.59 (0.06)^a^
No	80 (100)	78 (97.5)	2 (2.5)	
Not able to be determined	149 (100)	144 (96.6)	5 (3.4)	
Degree of urbanization of the CPs				
Cities	133 (100)	131 (98.5)	2 (1.5)	0.12 (0.12)^a^
Towns and semi‐dense areas	121 (100)	119 (98.3)	2 (1.7)	
Rural areas	64 (100)	60 (93.8)	4 (6.2)	
Purchasing power index by city/district of the CPs				
≤ 100%	183 (100)	176 (96.2)	7 (3.8)	0.15 (0.10)^b^
> 100%	135 (100)	134 (99.3)	1 (0.7)	

*Note:* *Significant at *p* < 0.05.

^a^ Fisher–Freeman–Halton exact test *p* value (Cramer's V).

^b^ Fisher exact test *p* value (Cramer's V).

## 4 Discussion

The high immediate availability of UPA preparations observed in this MC study is consistent with the results of the only German MC study conducted in the capital, Berlin [32]. In that study, UPA preparations were immediately available in 98.4% of the CPs. The immediate availability of LNG preparations determined here is even significantly higher than in Berlin (86.8%)—albeit against the background of the comparatively small sample. In addition, in this MC study, the eight CPs that did not have UPA preparations immediately available were able to procure them by the following day at the latest. This rather rapid procurement may be attributable to the legal requirement in Germany that full‐range pharmaceutical wholesalers ensure the continuous supply of CPs [45]. Nevertheless, due to their superiority over LNG preparations [7], an immediate availability of 100% should be targeted for UPA preparations. Therefore, UPA preparations should be included in the list of required medicines to be kept in stock [46], in order to ensure immediate availability in all CPs.

Even though nationwide MC studies are still lacking, the results obtained here can be compared with those of international MC studies in regions with an urban‐rural mix. Based on the few known MC studies in US states, UPA preparations—but as prescription‐only medicine (POM)—are hardly immediately available. An MC study in Hawaii found an immediate availability of UPA preparations of only 2.6% [18]. In Georgia, immediate availability was even below 1% [33]. By comparison, immediate availability in an MC study in Pennsylvania was somewhat higher at 5.2% [29], but still very poor. The latest MC studies for LNG preparations also show a lower immediate availability than the results determined here. In an MC study conducted in the US states of Arizona, Utah, New Mexico, and California, LNG preparations were immediately available in 80.6% of CPs [23]. In an MC study limited to California, immediate availability was 77.4% [27]. An MC study in Pennsylvania reported an immediate availability of 68.1% [29]. By contrast, immediate availability was only 57.7% in Georgia [33] and 47.4% in West Virginia [31], both of which were relatively low. In a Canadian MC study conducted in one province, immediate availability was comparatively high at 87.4% [25].

The high immediate availability of oral EC in German CPs supports the timely management of emergency situations. However, the relatively high prices of oral EC as an OTC medicine, compared with other OTC medicines, may hinder unrestricted access. In German SPM studies conducted as visits, the prices for OTC medicines were EUR 2.36 for acute diarrhea [47], EUR 3.46 [48], and EUR 4.95 [49] for non‐chronic tension‐type headache, and EUR 3.24 for nasal sprays for a common cold [50]. By contrast, in a German MC study, the prices were EUR 35.00 for UPA preparations and EUR 22.00 for LNG preparations [32]. Since these prices for oral EC are limited to the German capital Berlin, further SPM studies on pricing in other regions of Germany are warranted. Due to the small sample size, an additional nationwide MC study—with an LNG‐specific scenario—is recommended to obtain more meaningful information on immediate availability of LNG preparations. In addition, the availability of UPA preparations should be examined on a more localized level in future MC studies.

With regard to the possible influencing factors examined here, UPA preparations were more often not immediately available in rural areas and in areas with below‐average purchasing power, although these associations—similar to the other factors—were not statistically significant. In most international MC studies on the availability of oral EC, possible influencing factors tend to be understudied. However, the role of purchasing power, gender of the MCs, and urban‐rural setting can be compared, as these have been examined several times in international MC studies. Comparisons between CPs in low‐income and high‐income regions [16, 20, 26, 27, 30] and between female and male MCs [21, 28] revealed no significant differences. Likewise, no significant differences between rural and urban CPs were reported [19, 23, 24, 29]. In contrast, one MC study [33] and one SPM study conducted as visits [51] did find significantly higher immediate availability of LNG preparations in urban CPs, indicating a need for further research. In addition, several US MC studies have examined the influence of CP type (chain vs. independent CPs) (e.g., [19, 24, 27, 29, 33]). In Germany, however, such studies are limited due to the legal restriction that no more than four CPs may be owned (a maximum of three branches in addition to the main CP). Overall, future MC studies should investigate the relationship between the availability of oral EC and as many different influencing factors as possible.

## 5 Strengths and Limitations

This is the first nationwide study on the availability of oral EC by CPs in the world. However, against the background of the specific application of calls, the results do not contain any information on the availability of preparations actually recommended on site. The planned pharmacy‐specific performance feedback recommended in the international literature [52] had to be omitted due to project time constraints and limited human resources. Because some mystery calls were terminated prematurely by the pharmacy staff, the availability of oral EC could not be determined in all mystery calls. Therefore, the results are not representative of German CPs, as the minimum sample size was not reached. Nevertheless, the results—particularly those related to UPA preparations—provide a robust assessment.

## 6 Conclusion

Oral EC is very widely available immediately in Germany. However, for UPA preparations, the optimal immediate availability of 100% was not achieved, which may hinder access.

## Author Contributions

**Christian Kunow:** conceptualization, methodology, validation, writing – original draft preparation. **Carolin Priepke:** writing – review and editing. **Bernhard Langer:** conceptualization, data curation, formal analysis, methodology, project administration, validation, writing – review and editing. All authors read and approved the final manuscript.

## Funding

The authors received no specific funding for this work.

## Conflicts of Interest

The authors declare no conflicts of interest.

## 1 Transparency Statement

The lead author, Bernhard Langer, affirms that this manuscript is an honest, accurate, and transparent account of the study being reported; that no important aspects of the study have been omitted; and that any discrepancies from the study as planned (and, if relevant, registered) have been explained.

## Data Availability

The corresponding author had full access to all of the data in this study and takes complete responsibility for the integrity of the data and the accuracy of the data analysis. The data sets are available from the corresponding author upon reasonable request.
